# Diversity and Distribution of Archaea in the Mangrove Sediment of Sundarbans

**DOI:** 10.1155/2015/968582

**Published:** 2015-08-06

**Authors:** Anish Bhattacharyya, Niladri Shekhar Majumder, Pijush Basak, Shayantan Mukherji, Debojyoti Roy, Sudip Nag, Anwesha Haldar, Dhrubajyoti Chattopadhyay, Suparna Mitra, Maitree Bhattacharyya, Abhrajyoti Ghosh

**Affiliations:** ^1^Department of Biochemistry, University of Calcutta, 35 Ballygunge Circular Road, Kolkata, West Bengal 700019, India; ^2^Roche Diagnostics India Pvt. Ltd., Block 4C, Akash Tower, Near Ruby Hospital, 781 Anandapur, Kolkata 700107, India; ^3^Department of Biochemistry, Bose Institute, P1/12, C. I. T. Road, Scheme VIIM, Kolkata, West Bengal 700054, India; ^4^Department of Geography, University of Calcutta, 35 Ballygunge Circular Road, Kolkata, West Bengal 700019, India; ^5^Norwich Medical School, University of East Anglia and Institute of Food Research, Norwich Research Park, Norwich, Norfolk NR4 7UA, UK

## Abstract

Mangroves are among the most diverse and productive coastal ecosystems in the tropical and subtropical regions. Environmental conditions particular to this biome make mangroves hotspots for microbial diversity, and the resident microbial communities play essential roles in maintenance of the ecosystem. Recently, there has been increasing interest to understand the composition and contribution of microorganisms in mangroves. In the present study, we have analyzed the diversity and distribution of archaea in the tropical mangrove sediments of Sundarbans using 16S rRNA gene amplicon sequencing. The extraction of DNA from sediment samples and the direct application of 16S rRNA gene amplicon sequencing resulted in approximately 142 Mb of data from three distinct mangrove areas (Godkhali, Bonnie camp, and Dhulibhashani). The taxonomic analysis revealed the dominance of phyla Euryarchaeota and Thaumarchaeota (Marine Group I) within our dataset. The distribution of different archaeal taxa and respective statistical analysis (SIMPER, NMDS) revealed a clear community shift along the sampling stations. The sampling stations (Godkhali and Bonnie camp) with history of higher hydrocarbon/oil pollution showed different archaeal community pattern (dominated by haloarchaea) compared to station (Dhulibhashani) with nearly pristine environment (dominated by methanogens). It is indicated that sediment archaeal community patterns were influenced by environmental conditions.

## 1. Introduction

Archaea, representing the third domain of life, were originally anticipated to thrive under extreme environments, such as hydrothermal vents, hot water springs, salt brines, and extremely acidic and anoxic environments, where they contribute significantly towards the maintenance of the biogeochemical cycles [1–7]. However, with the advent of molecular techniques, it has become increasingly evident that archaea are much more widespread and metabolically diverse than originally postulated. A considerable portion of the microbial communities in a wide variety of “nonextreme” environments, for example, soil, ocean, and lakes, is constituted by archaea [8–13]. Despite the increasing interest to understand the ecophysiology of archaea, the lack of knowledge with respect to mesophilic and cold-adopted archaea is still enormous [9, 14, 15].

Mangrove wetlands are typical example of mesophilic and moderately halophilic environmental niches. They are commonly situated at the intertidal zones along the tropical and subtropical coasts and play a very important role in shaping the coastal ecology [16]. Mangrove forests are considered to be highly productive niche that support detritus-based food web [16, 17]. Particularly, in tropical mangroves, the high turnover rates for organic matters and nutrient cycling between the ocean and terrestrial habitats makes it the most productive ecosystem in the world [17]. The high primary productivity of mangroves implies a high demand for nutrients essential for plant growth and this appears to be achieved by a highly efficient system of nutrient trapping, uptake, and recycling in mangrove ecosystem [18]. The diverse microbial communities residing in the mangroves play important role in transformation of nutrients in the environment. While the importance of bacteria and fungi in mangrove biogeochemical cycles is well established, our knowledge of archaea in mangrove habitats remains extremely limited [16].

Sundarbans is the world's largest tidal halophytic mangrove ecosystem covering 20,400 square kilometers (7,900 sq mi) of area and has been recognized as a UNESCO World Heritage site. Situated in the delta of Ganges, Meghna, and Brahmaputra rivers on the Bay of Bengal, Sundarbans is shared between India and Bangladesh. This mangrove ecosystem is the home for diverse flora and fauna, including mangrove plant species like sundari (*Heritiera fomes*), goran (*Ceriops decandra*), geoa (*Excoecaria agallocha*), keora (*Sonneratia apetala*), and so forth, and the world's famous endangered royal Bengal tiger (*Panthera tigris tigris*). Microbial communities play an important role in generation of detritus in mangrove areas and Sundarbans is one such ecotype where microbes have been shown to be involved in biogeochemical cycling of the nutrients [18–20]. Despite global advancement in understanding the microbial diversity and role of microbes in different mangrove environments, little has been performed in Sundarbans [21, 22]. In a very recent study, a detailed description of the bacterial diversity has been presented in the backdrop of seasonal changes [23]. However, no data are yet available regarding the abundance and diversity of archaea in Sundarbans.

Hitherto, most of the research on mangrove environments has focused on understanding the diversity and functions of bacteria and fungi [23–26], and very little is known about archaeal assemblages in mangrove [16, 27, 28]. In order to gain new insight into the archaeal community patterns and the influence of environmental conditions in the mangrove sediments and to build foundational information for future research, we have investigated the archaeal diversity in the sediments of Indian Sundarbans employing 454-pyrosequencing.

## 2. Materials and Methods

### 2.1. Ethics Statement

No specific permits were required for the described field studies, which complied with all relevant regulation. The studied locations are not privately owned. Moreover, the study did not involve endangered or protected species. Indeed, the Indian Coastal Zone Management (ICZM) authority of the state of West Bengal, India, has approved this experimental exercise.

### 2.2. Study Area and Sediment Sampling

**Figure 1:**
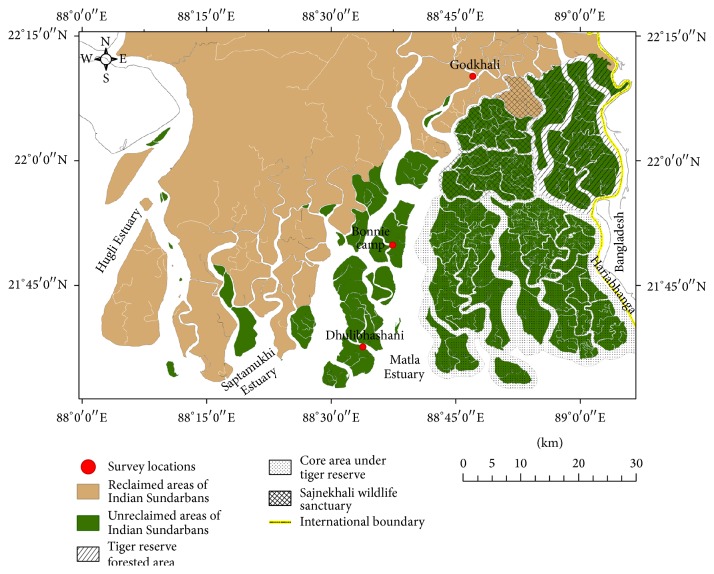
Geographical location of the sampling stations (Jharkhali-A, Sahidnagar-B, and Godkhali-C) in Indian Sundarbans. Coordinates of the sampling points and description of the stations are presented in Section 2.

Samples were collected in triplicate from surface (2 cm) and subsurface (32 cm) sediments of the Sundarbans mangrove wetland, which is located on the northeastern coast of India (Figure 1). The samples were collected from three different stations; for example, Godkhali (station A; 22°06′32.570′′N 88°46′22.220′′E), Bonnie camp (station B; 21°49′53.581′′N 88°36′44.860′′E), and Dhulibhashani (station C; 21°37′40.837′′N 88°33′47.762′′E) spanning 90 km along the tidal gradient from the shoreline (Figure 1). In June 2013, the sediment samples were collected in triplicate from each station. A sediment corer (50 cm depth, 6 cm diameter) was used to collect the top 32 cm of sediments. The uppermost surface layer (0–2 cm) and deeper subsurface layer (30–32 cm) were sampled and immediately sieved by a 2 mm mesh in the field. The sieved fractions were stored in sealed sterile polypropylene containers and brought to the laboratory. Upon arrival, a portion of each sediment sample was flash-frozen at −80°C for polyaromatic hydrocarbon (PAH) analysis and DNA extraction. The remaining sediments were stored at 4°C for other analytical procedures such as nutrients and heavy metals estimation. The individual collection from each sampling station was used for physicochemical analysis and in case of DNA extraction for sequencing analysis; three individual collections were mixed to homogeneity to generate a representative composite sample.

### 2.3. Sediment Analyses and Site Climate

Microbiological and biochemical analyses were performed with the field moist sediments. Physical and chemical analyses were carried out with air-dried sediment samples. The sediment pH was measured in 1 : 2.5 sediment-water suspensions and found to be alkaline. The total organic carbon (TOC) was measured by the methods described previously [29, 30]. Briefly, TOC in a sample was determined by combusting the air-dried sediment sample catalytically in oxygen atmosphere into instrument chamber at 500°–900°C and the resulting carbon dioxide gas was detected by a nondispersive infrared (NDIR) detector in an Aurora TOC Analyzer that was calibrated to directly display the detected carbon dioxide mass. This mass was proportional to the TOC mass in the sediment sample and calculated as total mass of carbon per unit of sediment sample.

Conductivity and salinity were measured* in situ* with Hach Portable Meters (HQ40d). Measured salinity was expressed in parts per thousand (ppt) or gm Kg^−1^ as described previously [31]. Nutrients like inorganic nitrogen (ammonia, nitrite, and nitrate), soluble phosphate, and reactive silicate were measured after quantitative extraction in respective buffering conditions following standard methodologies [32]. Briefly, nitrite was measured after complexing with sulphanilamide followed by a coupling reaction with n(1-napthyl)-ethylenediamene dihydrochloride, which forms an azo dye upon coupling. The resulting azo dye was measured spectrophotometrically at 543 nm. The nitrate, in contrary, was quantitatively reduced to nitrite using cadmium (Cd) granules prior to measurement. The total nitrite was then measured spectrophotometrically as described earlier and further subtraction of the measured value of free nitrite in the sediment resulted in determination of nitrate in the sample. Ammonia was measured in a reaction with hypochlorite under alkaline condition, which results in formation of monochloramine. In a successive reaction with phenol and nitroprusside, monochloramine was converted into indophenol blue, which was measured spectrophotometrically at 630 nm. The soluble phosphate was measured using acidified molybdate reagent, which yields phosphomolybdate complex upon reaction with soluble phosphate. This complex was further reduced into molybdenum blue and measured spectrophotometrically at 880 nm. The reactive silicate was measured using the formation of yellow silicomolybdic acid in presence of molybdate under acidic condition.

Organic pollutants (polyaromatic hydrocarbons, PAH) were measured using a combined gas chromatography and mass spectrometry (GC/MS) method described previously [33, 34]. Heavy metals in the sediment samples were assessed using atomic absorption spectrophotometric technique (Agilent Technologies, CA, USA).

### 2.4. Sediment DNA Isolation

For 454-pyrosequencing, each of the sediment samples from a station (total *n* = 3) and aliquots of homogenized sediment of 0.5 g were subjected to DNA extraction using the MoBio PowerSoil DNA Isolation Kit (MoBio Laboratories, Carlsbad, CA). After the extraction, DNA from all three samples from each sampling station was pooled together (approximately 200 ng of DNA from each extraction), and the pooled DNA was concentrated in a speed vacuum centrifuge (2,500 rpm, 30 min) to a final volume of 25 *μ*L (approximately 24 ng *μ*L^−1^). A NanoDrop (Thermo Scientific, Wilmington, DE, USA) spectrophotometer was used to quantify the extracted pooled DNA and to measure other important parameters for DNA quality, such as the ratio of absorbance at 260/280 nm and 260/230 nm.

### 2.5. PCR Amplification of Archaeal 16S rRNA Gene

To analyze archaeal diversity, the V3–V5 region of archaeal 16S rRNA gene was amplified by PCR. The PCR reaction (25 *μ*L) contained 5 *μ*L of 5-fold Phusion GC buffer (Finnzymes, Vantaa, Finland), 200 *μ*M of each of the four deoxynucleoside triphosphates, 1.5 mM MgCl_2_, 4 *μ*M of each primer (see Table S1 of the Supplementary Material available online at http://dx.doi.org/10.1155/2015/968582), 2.5% DMSO, 1 U of Phusion High Fidelity Hot Start DNA polymerase (Finnzymes), and 25 ng of pooled sediment DNA. The following thermal cycling scheme was used on a Veriti Thermal Cycler (Applied Biosystems, USA): initial denaturation at 98°C for 5 min, 25 cycles of denaturation at 98°C for 45 s, and annealing at 68°C for 45 s, followed by extension at 72°C for 30 s. The final extension was carried out at 72°C for 5 min. Negative controls were performed by using the reaction mixture without template. Primer sequences for amplification of the V3–V5 region [35] as well as 454 adaptors with the unique MIDs for each sample are listed in Table S1.

### 2.6. Library Preparation

PCR amplicons were evaluated by electrophoresis on a 1.5% agarose gel. The amplicon library was purified with Agencourt AMPure XP beads (Beckman Coulter Inc., Canada) and quantified by fluorometry using the Quant-iT PicoGreen dsDNA Assay Kit (Invitrogen, Burlington, ON) according to the Roche 454 “Amplicon Library Preparation Method Manual” of the GS Junior Titanium Series (454 Life Sciences, USA). Pooled amplicons were diluted as recommended and amplified by emulsion PCR on a Thermal Cycler 9700 (Applied Biosystem) according to the Roche 454 “emPCR Amplification Method Manual Lib-L” (454 Life Sciences, USA).

### 2.7. Sequencing and Data Processing

Pyrosequencing was performed for 200 cycles on a Roche 454 GS Junior sequencing instrument according to the manufacturer's protocol (454 Life Sciences, USA). All reads were filtered using the standard read rejecting filters of the GS Junior sequencer, namely, key pass filters, dot and mixed filters, signal intensity filters, and primer filters (454 Sequencing System Software Manual, V 2.5.3, 454 Life Sciences, USA). Raw sequencing and processing data was carried out using a combination of mothur (software version 1.28.0) and Ribosomal Database Project (RDP-II). The raw data were subjected to initial quality trimming using the mothur software and all reads having an average quality value of <20 were discarded. Then we removed the number of chimeric sequences by using UCHIME algorithm. Then the chimeric free reads were further screened for the presence of ambiguous bases and any reads which have a length <200 were discarded and forward primer sequence was removed from the final dataset. The obtained processed reads were then demultiplexed to separate sample based on the 10 bp MID sequence and any reads which do not match the MID were discarded. The high quality reads were then aligned to archaeal 16S rRNA sequences and clustering was performed at 97% similarity; using the RDP pipeline, a representative sequence from each cluster was selected based on abundance using the sequence selection tool. The representative sequences obtained from each cluster were then classified using the naïve Bayesian Classifier (Ribosomal Data Project, release 10) at a bootstrap confidence of 80.

### 2.8. Taxonomic Annotation

After quality trimming all the sequence reads were aligned against the SILVA, which is a curated ribosomal RNA sequence database using BLASTN [36, 37]. The resulting output files of read sequences were imported and analyzed using the paired-end protocol of MEGAN [38] to obtain taxonomic profiles as described earlier [39]. When processing the BLAST output files by MEGAN we used parameter settings of “Min Score = 35,” “Top Percent = 100,” “Min Support = 5,” and “Minimum Sequence Complexity Threshold = 0.44.” Reads which did not have any match to the respective database were placed under “No hit” node. Any reads that were originally assigned to a taxon that did not meet our selected threshold criterion were pushed back using the lowest common ancestor (LCA) algorithm to higher nodes where the threshold was met. After importing the datasets in MEGAN we obtained a set of MEGAN-proprietary “rma files” for each data mapped onto the NCBI taxonomy based on our selected threshold for tree visualization purpose.

All six RMA files were normalized to the smallest data set size (without including reads classified as not having a taxonomic assignment) to allow intersample comparison of taxonomic abundances and to obtain comparative tree view for all samples. Comparative abundance of read counts at different levels of NCBI taxonomy (class, order, family, genus, and species) was exported from all sample comparison-file for later statistical analyses.

### 2.9. Statistical Analyses

Hierarchical cluster analyses have been performed using R 3.0.2 [40], with species abundance data where Pearson's correlation is used for clustering the probes/genes (rows) and Spearman rank correlation is used for clustering the sample datasets (cols), with complete linkage.

Composition (presence/absence) data were also analyzed to compare the community structure. SIMPER analyses were used to determine which taxa/function contributed most to the observed differences. Further data were analyzed using common multivariate ordination techniques: Nonmetric Multidimensional Scaling (NMDS) using Bray Curtis distance. To understand the influence of physicochemical parameters in different sampling stations, Principal Component Analysis (PCA) was performed. Additionally, we have performed correspondence analyses on genomic data as well as on PAH (polyaromatic hydrocarbons) data.

We have also plotted the genomic data using Voronoi diagram, which is a partitioning of a plane into regions based on distance to points in a specific subset of the plane. In this plot we have highlighted 6 major phyla.

### 2.10. Nucleotide Sequence Accession Numbers

All 454-GS Junior sequence data from this study were submitted to the NCBI Sequence Read Archive (SRA) under accession numbers SRR1632262 (Godkhali_surface), SRR1632263 (Godkhali_subsurface), SRR1632258 (Bonnie camp_surface), SRR1632259 (Bonnie camp_subsurface), SRR1632260 (Dhulibhashani_surface), and SRR1632261 (Dhulibhashani_subsurface).

## 3. Results

### 3.1. Environmental Parameters

Physicochemical characteristics such as pH, dissolved oxygen, saturation, total carbon, total nitrogen, and salinity were estimated for surface and subsurface samples from all the three stations in the present study (Table 1 and Table S2). The environmental parameters, for example, salinity, temperature, dissolved oxygen, pH, and saturation were measured* in situ* (Table 1). Temperature and salinity ranged from 29.7 to 32.8°C and from 21 to 22.8 psu, respectively. The lowest temperature and highest salinity were recorded in Dhulibhashani.

Measurement of NO_2_^−^, NO_3_^−^, and NH_4_^+^ in the surface and subsurface samples from all the three sampling stations revealed a differential pattern for these nitrogen species (Table S2). Ammonia was found maximum in the sediment samples of Godkhali, while it was minimum in the sediment of Dhulibhashani (*P* < 0.05). Nitrite was found negligible in all the stations confirming very high rate of nitrification, resulting in nitrate formation, which was evenly detected in all three sampling stations (Table S1). Other parameters such as phosphate, sulphate, and silicate were found evenly distributed in all the sampling stations possibly due to regular inundation.

Heavy metals have been shown to play a pivotal anthropogenic role in the marine environment. Previously, it has been shown that heavy metals like Fe, Zn, Cu, Mn, Hg, Pb, Ni, Cr, and Cd are present in the Sundarbans estuaries [41–44]. To ascertain the global ecological impact of different heavy metals in the present study, concentrations of heavy metals like Fe, Cd, Zn, Cu, Ni, and Pd were measured in surface and subsurface samples collected from all the three stations (Table S2). Heavy metal estimates for the Sundarbans sediment found in our study were fairly consistent with previously found results [45].

### 3.2. Archaeal Community Structure Revealed by 16S rRNA Gene Based Analysis

**Figure 2:**
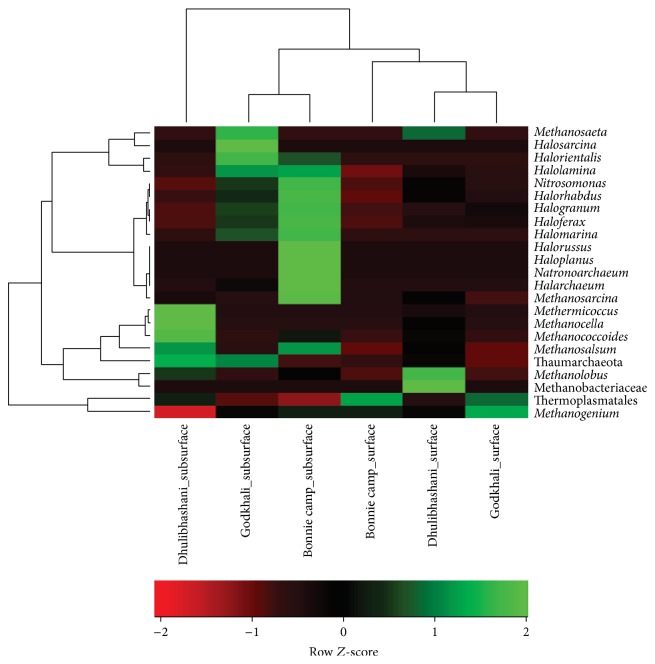
Hierarchical clustering (complete-linkage) heat map generated from taxonomic abundance profile (using Spearman's rank correlation) reflecting spatial distribution of archaeal class in the sediment of Sundarbans (using Pearson's correlation).

We analyzed archaeal 16S rRNA gene (SSU) amplicons prepared from six sediment samples originated from three sampling stations at two different depths. After quality filtering, denoising, and removal of potential chimeric sequences of pyrosequencing dataset, this resulted in recovery of 1,41,816 sequences with an average length ranges between 500 and 510 bases (506 bp average) (Table 2). Within our sequence dataset, we were able to assign 1,39,953 sequences to the domain archaea and to classify all of these sequences below domain level using BLASTN 2.2.25+ in SILVA database and MEGAN (Table 2). The classified sequences were affiliated to two archaeal phyla and five archaeal classes or similar phylogenetic group (Figure 2 and Figure S1). Euryarchaeota often was the most abundant phylum (36–60%) and Thermoplasmata was the predominant class across all samples (39–62%) (Figure S1). Besides Euryarchaeota, Thaumarchaeota (Marine group I) was found to be highly abundant in all the samples (Table S3). Within euryarchaeal sequences, a number of members of the class Halobacteria, for example,* Halosarcina*,* Halorientalis*,* Halolamina*,* Halorhabdus*,* Halogranum*,* Haloferax*,* Halomarina*,* Halorussus*,* Haloplanus*, and* Halarchaeum*, and of the class Methanomicrobia, for example,* Methanosarcina*,* Methermicoccus*,* Methanocella*,* Methanococcoides*,* Methanosalsum*,* Methanolobus*, and* Methanogenium* were detected (Figure 2). Besides, few sequences were also affiliated to classes Thermoplasmata and Methanobacteria. Unfortunately, within our datasets, we could not detect any representative of the archaeal phyla Crenarchaeota, Korarchaeota, Nanoarchaeota, and Nanohaloarchaeota due to lower coverage of the degenerate primers used in this study.

### 3.3. Diversity and Species Richness of Archaeal Communities

A diversity index is a mathematical measure of species diversity in a community. Diversity indices provide important information about community composition rather than only species richness (i.e., the number of species present); and simultaneously they also take the relative abundances of different species into account. We evaluated the archaeal diversity and evenness using the normalized dataset. Considering all the sampling sites, the Shannon-Weaver (*H*′) index varied from 0.67 to 1.08 and the Simpson diversity (1 − *λ*′) index varied from 0.29 to 0.52 (Table 3). Such variations of diversity indicated that the archaeal diversity is higher at the surface samples both in Bonnie camp and Godkhali. However, in Dhulibhashani, archaeal diversity was found to be almost similar both at the surface and at subsurface level with a marginally higher diversity in subsurface sample. The Pielou index (*J*′) was between 0.23 and 0.37 and the Margalef (*d*) index was between 1.52 and 3.69 (Table 3). Diversity and evenness are more informative for describing community composition than simple phylotype richness levels. Community diversity, as reflected by the Shannon index, was highest in Bonnie camp_subsurface and lowest in Godkhali_subsurface and is by definition generally correlated positively with the number of unique phylotypes and/or with greater community evenness.

### 3.4. Taxonomic Assignments and Statistics

In addition to surveying archaeal diversity at the surface and subsurface sediments of three sites in Sundarbans, pyrosequencing also allowed assessment of the relative abundance of the taxonomic levels of archaea detected. A total of 23 genera (genus/order/family) were commonly shared between six samples (Figure 2 and Figure S1). Interestingly, the dominant class/phylum showed some geographical characteristics. For example, Halobacteria were highly abundant in the subsurface layers of the representative samples from Godkhali and Bonnie camp. In contrary, Methanomicrobia were only dominant in the subsurface sediment of Dhulibhashani.

Cluster analysis showed that the archaeal community detected in the subsurface sediment of Dhulibhashani was the most dissimilar of all the sediment samples tested (Figure 2). Additionally, among the subsurface sediments, the most diverse archaeal community was detected in this sediment. Among others, there is a clear separation of two types of samples, surface and subsurface. The surface sediments have shown an overall similarity among Godkhali and Bonnie camp while Dhulibhashani surface showed a different community profile.

**Figure 3:**
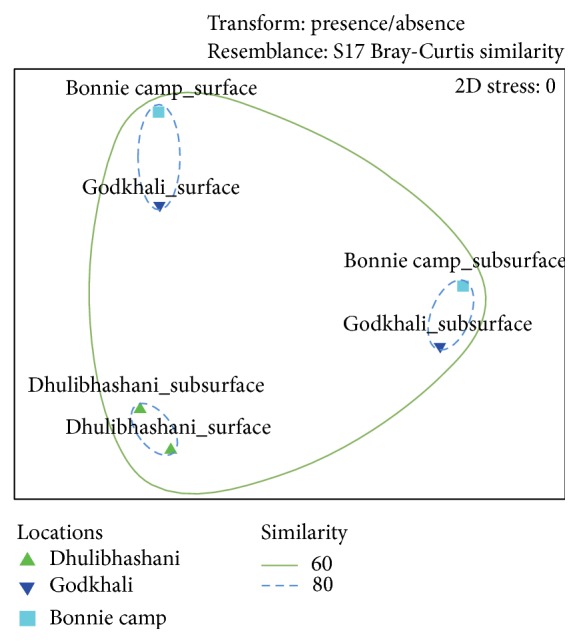
Archaeal community compositional structure in the sediments of Sundarbans indicated by Nonmetric Multidimensional Scaling (NMDS) using Bray-Curtis distance. Gene abundance data was transformed based on presence/absence before creating Bray-Curtis resemblance matrix for NMDS analysis (showing similarity based on community composition only).

Further we have performed Nonmetric Multidimensional Scaling (NMDS) to compare the community composition using presence/absence data. NMDS employs an iterative algorithm that optimizes the position of samples into a low-dimensional space minimizing the difference between rank-order of multivariate ecological dissimilarity and distances in NMDS space (Figure 3). From the figure it is easily observed that despite the presence of very few abundant taxa in more than one sample, surface and subsurface communities from different sampling stations or locations (Godkhali and Bonnie camp) were more closely related to each other than to samples from the same location. Samples from Dhulibhashani show similar results to cluster analyses, showing much different community structure than the other locations for both surface and subsurface samples. To this end we believe that the presence or absence of certain taxonomic groups in the sediment of Dhulibhashani is probably due its proximity to the open ocean (Bay of Bengal) and the nutritional status of the sediment.

**Figure 4:**
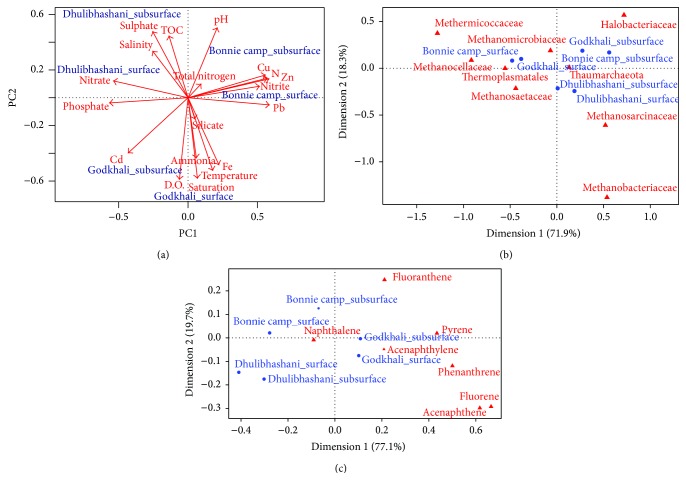
(a) Principal Component Analysis (PCA) of samples based on environmental parameters, nutrient, and heavy metal levels in the sediments samples. (b) Correspondence analysis (CA) based on distribution of major taxonomic lineages generated analyzing genomic data in the sediment samples. (c) Correspondence analysis (CA) based on distribution of polyaromatic hydrocarbons (PAH) in the sediment samples.

Furthermore, our PCA analysis revealed that the physicochemical parameters influence sampling stations differentially. The first PCA axis showed high positive correlations with heavy metals Zn, Pb, and Cu (Figure 4(a)). Samples from Bonnie camp showed high positive correlations with all of these heavy metals. In contrary, samples from Godkhali showed positive correlations with heavy metals Cd and Fe (Figure 4(a)). To this end, PCA analysis revealed that both of these sampling stations at Godkhali and Bonnie camp were at an elevated risk compared to Dhulibhashani, regarding heavy metal pollution. Notably, these two sampling stations also showed positive correlations with pH, DO, silicate, nitrite, ammonia, saturation, and total nitrogen (Figure 4(a)). Dhulibhashani, on the other hand, showed positive correlations with TOC, nitrate, sulphate, and salinity (Figure 4(a)).

Further, we have performed correspondence analysis (CA) to compare the influence of abundant taxonomic groups and polyaromatic hydrocarbons (PAH) in these sampling sediments (Figures 4(b) and 4(c)). The correspondence analysis comparing abundant taxonomic groups in the sampling stations clearly indicated that the subsurface sediments from Godkhali and Bonnie camp were heavily dominated by Halobacteriaceae (Figure 4(b)), whereas surface archaeal communities in these sampling stations were dominated by Methanomicrobiaceae, Methermicoccaceae, and Methanocellaceae (Figure 4(b)). Both the surface and subsurface archaeal communities in Dhulibhashani were heavily dominated by Methanosarcinaceae and Methanobacteriaceae (Figure 4(b)). To summarize, methanogens were commonly encountered in the surface sediments of all three stations. However, while Halobacteriaceae dominated the subsurface archaeal population in Godkhali and Bonnie camp, methanogenic Methanosarcinaceae and Methanobacteriaceae dominated the subsurface sediment of Dhulibhashani.

Correspondence analysis, performed to understand the influence of PAHs in the sediments of the sampling stations, revealed that both the surface and subsurface sediments of Godkhali and Bonnie camp correlated with the detected PAH congeners (Figure 4(c)). In contrary, surface and subsurface sediments of Dhulibhashani showed little correlation to any of the identified PAHs. To this end, we believe that the pattern of correlation of PAHs in the sediments of Godkhali and Bonnie camp is in agreement with the detection of Halobacteriaceae, the most active hydrocarbon degrading archaeal representative, in the subsurface sediments of these stations. In general, hydrocarbons, by virtue of being hydrophobic in nature, are scarcely soluble in water and tend to adsorb readily onto organic matter, particularly sediments. In an environment like Sundarbans, surface sediments are in general more dynamic due to regular inundation. The hydrocarbons, therefore, remained mostly absorbed in the sediments and our data clearly shows that subsurface sediments contain more PAHs compared to surface sediments.

**Figure 5:**
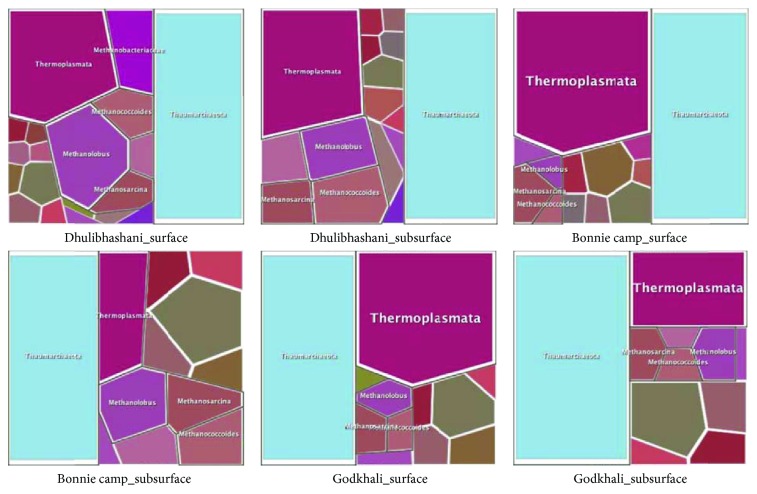
Voronoi diagram depicting comparative taxonomic profile composition pattern for each sample as a partition of a plane highlighting 6 major phyla.

Finally, we have generated Voronoi plot (Figure 5), which describes the proportionate taxonomic composition pattern for each sample as a partition of a plane.

### 3.5. Multivariate SIMPER (Similarity Percentage) Analysis

To reveal differences between three sampling stations, Godkhali, Bonnie camp, and Dhulibhashani, and to identify major taxonomic groups that contributed to the similarities or dissimilarities, SIMPER (Similarity Percentage) analysis was performed (Tables 4(a), 4(b), and 4(c)). At the regional scale, there were differences observed at order/family/genus level between three sampling stations. Overall, distribution of taxa was found similar in Godkhali and Bonnie camp with Thermoplasmatales being the major contributor towards the similarity (23.3%), and with only exception of* Methanosarcina*, the relative abundance of which was higher in Bonnie camp. In general, SIMPER analysis targeting relative abundance of taxa in different stations revealed higher relative abundance of class Halobacteria in Godkhali and Bonnie camp, while class Methanomicrobia was found relatively more abundant in Dhulibhashani (Tables 4(b) and 4(c)). Representatives of the class Methanomicrobia, such as* Methanolobus*,* Methanococcoides*,* Methermicoccus*, and* Methanocella*, were either higher or only present in Dhulibhashani. In contrary, representatives of class Halobacteria such as* Halogranum*,* Halorientalis*,* Haloferax*, and* Halarchaeum* were either higher or only present in Godkhali/Bonnie camp (Table 4(a)). Putative ammonia-oxidizing archaea in the phylum Thaumarchaeota were highly abundant in all three sampling sites. To our surprise, the order Thermoplasmatales representing acidophilic Euryarchaeota were highly abundant in all three sampling stations despite the fact that the recorded pH fell between 7.85 and 7.98 in these sites.

## 4. Discussion

In marine environments, microorganisms play an important role in sustainable maintenance of the ecosystem. They are key mediators of global biogeochemical cycling of the essential elements in the marine environments. Recent advancement of molecular microbial ecology has emerged as multiple studies on the diversity and abundance of microorganisms in these habitats and their role in shaping the sustainable ecosystem. Most of these studies focused on marine bacteria and archaea, whereas little is known on the diversity and ecology of mangrove archaea [16, 17, 23, 24]. Interestingly, archaea have been found to be ubiquitous and abundant members of the microbiome in diverse marine environments including coastal waters [46, 47], marine sediments, estuaries [48–50], stratified basins [7, 51], mangrove sediments [52–54], and open ocean water columns [47, 55]. In marine habitat archaea play crucial roles in nitrification [54, 56, 57], sulfur metabolism [58], methane oxidation (ANME) [59], and methanogenesis [60].

The present study focused on accessing the archaeal community structure and diversity in the world's largest tropical mangrove sediments of Sundarbans using 16S rRNA gene-based 454-amplicon sequencing. To our knowledge, this is the first study on understanding archaeal diversity in Sundarbans ecosystem. The majority of sequences obtained were affiliated to the Euryarchaeota. Previous studies by Sapp et al. on marine sediment and Wemheuer et al. on German Bight found high abundance of members of Euryarchaeota [35]. We identified Thermoplasmatales as the most abundant euryarchaeal group in the investigated samples. Most sequences were affiliated to the MKCST-A3, CCA47, and VC2.1Arc6 groups. The MKCST-A3 group was first identified in the mangrove sediment of China and most of the members reported so far are uncultured archaeon [16]. The CCA47 group was originally identified by 16S rRNA gene analysis of oxygen-depleted marine environment and anoxic subsaline sediments. The number of sequences within this group was also affiliated to the Marine Group II (Euryarchaeota). Previous studies by DeLong and Karl have suggested that the members of Marine Group II are more abundant in temperate sea water than Marine Group I (*Thaumarchaeota*) [61]. In our case, however, overall abundance of Marine Group I (Thaumarchaeota) is higher compared to Marine Group II (Euryarchaeota) possibly emphasizing geographical differences between tropical and temperate marine sediment microbial community. Moreover, this might also be due to the tidal current that influences the microbial community structure within Sundarbans sediment. The regular inundation of the subtidal zones within Sundarbans whirls up microbial cells to the water column. An in-depth analysis of the archaeal diversity and abundance in the water column might be useful to know whether we could detect a large number of sequences affiliated to Marine Group II. Unfortunately, only limited studies have been performed targeting archaeal communities in the water column in tropical mangrove. Due to such knowledge gap, the habitat preference of Marine Group II cannot be addressed properly at this time. The Thermoplasmatales cluster VC2.1Arc6 was originally described within the microbial community obtained from* in situ* growth chamber placed on a deep-sea hydrothermal vent on the Mid-Atlantic Ridge [62]. Besides Thermoplasmatales, the number of euryarchaeal sequences was affiliated to Halobacteria. Members of this group can grow aerobically as well as anaerobically. The majority of the halobacterial sequences analyzed in the present study were affiliated to the* Halogranum* genus. Besides, a significant number of sequences affiliated to* Natronomonas*,* Haloferax*,* Halorhabdus*, and* Halolamina* were identified. Methanomicrobia and Methanobacteria were the other two abundant euryarchaeal classes in the investigated samples. Methanomicrobia and Methanobacteria are known for their putative importance in sulfate reduction and methanogenesis in anoxic marine sediments, such as mangroves. The members of these classes are methanogens and are involved in carbon-cycle through methanogenesis.

Beside euryarchaeota, another archaeal group found in all samples was the Marine Group I (MG1) or Thaumarchaeota. Marine Group I (MGI) Thaumarchaeota are one of the most abundant and cosmopolitan chemoautotrophs and prokaryotic picoplankton within the global deep sea water. They were originally identified by sequencing of environmental 16S rRNA genes derived from sea water. All organisms of this lineage thus far identified are chemolithoautotrophic ammonia-oxidizers and may play important roles in biogeochemical cycles, such as the nitrogen cycle and the carbon cycle. Within our sequence dataset, the number of ammonia oxidizing Thaumarchaeota was identified.

We observed an increased abundance of Halobacteriaceae in the investigated subsurface samples from Godkhali and Bonnie camp. In contrary, the number of Methanosarcinaceae and Methanobacteriaceae was higher in the samples derived from Dhulibhashani. This might be correlated with the high amounts of organic matter in resident algal blooms in Godkhali and Bonnie camp (Figures 4(b) and 4(c)). Furthermore, correspondence analysis revealed that detected PAHs were mostly correlated to the surface and subsurface sediments of Godkhali and Bonnie camp. Previous studies from our group have shown that in Godkhali and Bonnie camp, because of the presence of jetty for transportation, large amount of hydrocarbon-derived chemicals and oils are released in water and in turn increase algal blooms in surrounding areas (Personal Communication). To this end, we believe that abundance of Halobacteriaceae in Godkhali and Bonnie camp corroborates well to inflated detection PAHs in the sediment. In hypersaline environment, Halobacteria are the most active organisms capable of organic matter degradation. Thus the higher abundance of Halobacteria in Godkhali and Bonnie camp might indicate an involvement in marine organic matter degradation under high nutrient conditions found during algal blooms. Similar results were reported by Teeling et al., where they demonstrated that bacterial community structures were highly influenced by the presence of an algal bloom [63]. Furthermore, Wemheuer et al. have recently demonstrated that archaeal diversity was influenced by algal blooms in German Bight [35]. Taken together, present observation indicates that marine microbial communities are influenced by algal blooms or by environmental parameters correlated with bloom presence.

## 5. Conclusions

In summary, the spatial variations of the sedimentary archaeal diversity have been studied in the Sundarbans mangrove ecosystem for the first time by means of 16S rRNA 454-pyrosequencing. We found highly diverse archaeal communities in the sediment of Sundarbans. Our analyses have confirmed the influence of environment in shaping the archaeal community diversity within the sediment. However, due to the lack of pure cultures and large comparative investigations, robust conclusions related to the involvement of identified archaeal communities in biogeochemical cycle cannot be drawn. Further research including analyses of expression of functional genes and determination of the active archaeal population within the sediment might unravel the role of archaea in Sundarbans mangrove ecosystem.

## Supplementary Material

Supplementary Figure 1: A phylogenetic tree was constructed with the assigned archaeal 16S rRNA sequences found in this study using MEGAN5 metagenome analyzer. The Euryarchaeota and Thaumarchaeota were the dominant representative phyla present within our dataset. In the Euryarchaeota phylum, Thermoplasmatales and Halobacteriaceae grouped the majority of the representative sequences of different samples.
Supplementary Table 1: Primers used for amplification of the V3–V5 region of the archaeal 16S rRNA.
Supplementary Table 2: Assessment of physico-chemical parameters, heavy metals and polyaromatic hydrocarbons (PAH) in Sundarbans sediments.
Supplementary Table 3: Comparative percent detection of Euryarchaeota and Thaumarchaeota in Sundarbans.

## Figures and Tables

**Table 1:** Environmental parameters of sampling sites analyzed in this study.

Sample	Site	Latitude °N	Longitude °E	Depth (cm)	*T* (°C)^*∗*^	Salinity (psu)^*∗*^	pH^*∗*^	DO (mg/L)^*∗*^
Surface	Godkhali	22°06′32.570′′	88°46′22.220′′	2	31.30	21.00	7.88	7.32
Subsurface	Godkhali	22°06′32.570′′	88°46′22.220′′	32	31.80	20.80	7.85	7.24
Surface	Bonnie camp	21°49′53.581′′	88°36′44.860′′	2	32.10	21.60	7.98	7.15
Subsurface	Bonnie camp	21°49′53.581′′	88°36′44.860′′	32	32.50	21.40	7.97	7.05
Surface	Dhulibhashani	21°37′40.837′′	88°33′47.762′′	2	29.70	22.80	7.96	7.15
Subsurface	Dhulibhashani	21°37′40.837′′	88°33′47.762′′	32	30.10	22.10	7.95	7.02

^*∗*^Average of three independent measurements, *n* = 3.

**Table 2:** Sample statistics.

Samples	Total number of reads sequenced	Assigned against SILVA using BLASTN 2.2.25+ and MEGAN
Godkhali_surface	15,939	15,647
Godkhali_subsurface	23,437	23,091
Bonnie camp_surface	15,371	15,120
Bonnie camp_subsurface	15,788	15,654
Dhulibhashani_surface	33,771	33,395
Dhulibhashani_subsurface	37,510	37,046

**Table 3:** Univariate diversity indices.

Sample	Total species (*S*)	Total individuals (*N*)	Species richness (Margalef): *d* = (*S* − 1)/Log(*N*)	Pielou's evenness *J*′ = *H*′/Log(*S*)	Shannon *H*′ = −SUM(*P*_*i*_ *∗*Log(*P*_*i*_))	Simpson (1 − Lambda′) = 1 − SUM(*N*_*i*_ *∗*(*N*_*i*_ − 1)/*N∗*(*N* − 1))
Godkhali_surface	11	100	2.171	0.3447	0.8264	0.5206
Godkhali_subsurface	17	100	3.474	0.2384	0.6753	0.2998
Bonnie camp_surface	8	100	1.52	0.3597	0.748	0.5104
Bonnie camp_subsurface	18	100	3.692	0.3763	1.088	0.4389
Dhulibhashani_surface	16	100	3.257	0.3454	0.9575	0.4761
Dhulibhashani_subsurface	13	100	2.606	0.357	0.9157	0.4858

**Table:** (a) Godkhali versus Bonnie camp

Taxon^1^	Order/family/genus	Contribution^2^ (%)	Average^3^ % abundance in Godkhali	Average % abundance in Bonnie camp	Average dissimilarities
Thermoplasmata	Thermoplasmatales	23.30	4.85	4.64	5.23
Halobacteria	*Halogranum *	11.72	1.50	1.64	2.63
Thaumarchaeota	Thaumarchaeota	10.08	8.29	7.99	2.26
Methanomicrobia	*Methanolobus *	8.33	0.65	0.99	1.87
Methanomicrobia	Methanococcoides	6.46	0.40	0.82	1.45
Halobacteria	*Haloferax *	5.42	0.48	0.47	1.22
Halobacteria	*Natronomonas *	5.41	0.62	0.73	1.21
Methanomicrobia	*Methanosarcina *	5.00	0.55	1.03	1.12
Halobacteria	*Halorhabdus *	3.97	0.35	0.35	0.89
Halobacteria	*Halolamina *	3.49	0.38	0.30	0.78
Methanomicrobia	*Methanosalsum *	3.46	0.13	0.33	0.78
Halobacteria	*Halarchaeum *	3.17	0.08	0.30	0.71
Halobacteria	*Halorientalis *	2.17	0.19	0.16	0.49

**Table:** (b) Dhulibhashani versus Godkhali

Taxon^1^	Order/family/genus	Contribution^2^ (%)	Average^3^ % abundance in Dhulibhashani	Average % abundance in Godkhali	Average dissimilarities
Methanomicrobia	*Methanolobus *	19.80	2.34	0.65	4.28
Thermoplasmata	Thermoplasmatales	18.75	4.59	4.85	4.05
Methanomicrobia	Methanococcoides	12.14	1.43	0.40	2.62
Thaumarchaeota	Thaumarchaeota	9.64	8.29	8.29	2.08
Halobacteria	*Halogranum *	8.80	0.75	1.50	1.90
Methanomicrobia	*Methermicoccus *	4.02	0.34	0.00	0.87
Methanomicrobia	*Methanosalsum *	3.84	0.45	0.13	0.83
Halobacteria	*Haloferax *	3.58	0.18	0.48	0.77
Halobacteria	*Natronomonas *	3.49	0.42	0.62	0.75
Methanomicrobia	*Methanocella *	2.47	0.21	0.00	0.53
Halobacteria	*Halorientalis *	2.20	0.00	0.19	0.47
Halobacteria	*Halolamina *	1.90	0.24	0.38	0.41

**Table:** (c) Dhulibhashani versus Bonnie camp

Taxon^1^	Order/family/genus	Contribution^2^ (%)	Average^3^ % abundance in station A	Average % abundance in station B	Average dissimilarities
Thermoplasmata	Thermoplasmatales	20.76	4.59	4.64	5.12
Methanomicrobia	*Methanolobus *	14.23	2.34	0.99	3.51
Halobacteria	*Halogranum *	10.05	0.75	1.64	2.48
Methanomicrobia	Methanococcoides	8.65	1.43	0.82	2.13
Thaumarchaeota	Thaumarchaeota	6.72	8.29	7.99	1.66
Halobacteria	*Natronomonas *	4.73	0.42	0.73	1.17
Halobacteria	*Haloferax *	4.42	0.18	0.47	1.09
Methanomicrobia	*Methanosarcina *	3.96	0.70	1.03	0.98
Methanomicrobia	*Methermicoccus *	3.42	0.34	0.00	0.84
Methanomicrobia	*Methanosalsum *	3.40	0.45	0.33	0.84
Halobacteria	*Halorhabdus *	3.37	0.24	0.35	0.83
Halobacteria	*Halolamina *	2.92	0.24	0.30	0.72
Halobacteria	*Halarchaeum *	2.70	0.00	0.30	0.67
Methanomicrobia	*Methanocella *	2.10	0.21	0.00	0.52

^1^Phylum or class level for archaea.

^2^Contribution of each taxon to the overall dissimilarity between these two clusters.

^3^Average abundance of each taxon in the two clusters.
