# Supplementary material for: Diversity and Distribution of Archaea in the Mangrove Sediment of Sundarbans
Source: Archaea. 2015 Aug 6;2015:968582. doi: 10.1155/2015/968582 (PMC4543378; doi:10.1155/2015/968582)
Supplement: Supplementary file 1 — Supplementary Figure 1: A phylogenetic tree was constructed with the assigned archaeal 16S rRNA sequences found in this study using MEGAN5 metagenome analyzer. The Euryarchaeota and Thaumarchaeota were the dominant representative phyla present within our dataset. In the Euryarchaeota phylum, Thermoplasmatales and Halobacteriaceae grouped the majority of the representative sequences of different samples. Supplementary Table 1: Primers used for amplification of the V3–V5 region of the archaeal 16S rRNA. Supplementary Table 2: Assessment of physico-chemical parameters, heavy metals and polyaromatic hydrocarbons (PAH) in Sundarbans sediments. Supplementary Table 3: Comparative percent detection of Euryarchaeota and Thaumarchaeota in Sundarbans. [file 968582.f1.zip › Supplementary Files/Table S1.docx]

| Sample | Primer | 454-Adaptor (Lib-L kit) | Sequence (5’-3’)  Key Unique MID | | Archaeal 16S rRNA specific |
| --- | --- | --- | --- | --- | --- |
| Surface | ARC344F | CCATCTCATCCCTGCGTGTCTCCGAC | TCAG | CTCGCGTGTC | ACGGGGYGCAGCAGGCGCGA |
| Subsurface | ARC344F | CCATCTCATCCCTGCGTGTCTCCGAC | TCAG | TCTCTATGCG | ACGGGGYGCAGCAGGCGCGA |
| All | ARC915R | CCTATCCCCTGTGTGCCTTGGCAGTC | TCAG | ACAGTATATA | GTGCTCCCCCGCCAATTCCT |

Table 1. Primers used for amplification of the V3–V5 region of the archaeal 16S rRNA
